# The Mediating Role of Body Mass Index in the Association Between Dietary Index for Gut Microbiota and Biological Age: A Study Based on NHANES 2007–2018

**DOI:** 10.3390/nu16234164

**Published:** 2024-11-30

**Authors:** Shuli An, Jian Qin, Xinjie Gong, Shuangshuang Li, Haiyan Ding, Xue Zhao, Hongqi He, Linwei Zhou, Xinrui Deng, Xia Chu

**Affiliations:** 1 Department of Nutrition and Food Hygiene, School of Public Health, Key Laboratory of Precision Nutrition and Health, Ministry of Education, Harbin Medical University, Harbin 150081, China; 202301094@hrbmu.edu.cn (S.A.); 2022020159@hrbmu.edu.cn (J.Q.); 2023020238@hrbmu.edu.cn (X.G.); 2022020237@hrbmu.edu.cn (S.L.); 2022020149@hrbmu.edu.cn (H.D.); 202201064@hrbmu.edu.cn (X.Z.); 2023020239@hrbmu.edu.cn (H.H.); 2023020160@hrbmu.edu.cn (L.Z.); 2 College of Food Science, Northeast Agricultural University, Harbin 150030, China; dxr-005@163.com

**Keywords:** diet, gut microbiota, biological age, body mass index, mediation, NHANES

## Abstract

Objective: The dietary index for gut microbiota (DI-GM) is a newly proposed metric for assessing diet quality, and its relationship with biological age is unclear. We hypothesize that consuming foods conducive to a healthy gut microbiota environment may decelerate aging. Methods: This cross-sectional study utilized data from the National Health and Nutrition Examination Survey (NHANES) spanning the years 2007 to 2018. The DI-GM was calculated by averaging the intakes from two 24-h dietary recall interviews. The biological age indicators were assessed using the Klemera–Doubal Method (KDM), phenotypic age (PA), and homeostasis disorder (HD). Logistic regression, restricted cubic splines (RCS), and mediation analysis were employed to explore the association between DI-GM and KDM, PA, and HD. Results: The study included 20,671 participants. According to the logistic regression model, adjusting for all covariates, a negative association was observed between the DI-GM score and biomarkers of biological aging. Compared to participants in the lowest quartile for DI-GM scores, those in the highest quartile exhibited reduced odds ratio (OR) for all of the biological age indicators, namely biological age assessed via KDM (OR: 0.69, 95% CI: 0.60–0.79), PA (OR: 0.84, 95% CI: 0.73–0.97), and HD (OR: 0.86, 95% CI: 0.76–0.98). Additionally, RCS analysis revealed a nonlinear association between DI-GM and biological age. Mediation analysis showed that the body mass index (BMI) partly mediated the association between DI-GM and biological age. Conclusions: Therefore, we concluded that a higher DI-GM score is associated with a lower risk of accelerated aging, with BMI mediating this association. Future research should validate these findings through the use of longitudinal studies.

## 1. Introduction

Aging is a complex physiological process that is irreversible and caused by a variety of factors. It is characterized by the gradual loss of physiological integrity, the accumulation of molecular changes, the decline of physiological functions, and, ultimately, death [1,2]. Currently, the world is facing increasingly serious challenges related to aging. It is projected that by 2050, the population aged 60 years and over will increase to 2.1 billion [3]. Aging exacerbates the burden of chronic disease, increases socio-economic costs, and raises the risk of adult mortality from disease [4]. Numerous studies have confirmed the strong association between aging and a wide range of diseases such as cancer, hypertension, diabetes, and cardiovascular disease (CVD) [5,6,7]. Therefore, effectively slowing down aging has become a key issue in public health. Utilizing machine learning algorithms to analyze multiple biomarkers in blood, it is possible not only to assess the degree of biological aging but also to reveal potential risks of accelerated aging beyond chronological age, providing us with a more accurate picture of an individual’s physiological aging condition [8]. At present, the most common indicators for evaluating biological age are the Klemera–Doubal Method (KDM), phenotypic age (PA), and homeostasis disorder (HD) [2,9,10]. KDM represents the chronological age at which an individual’s physiology is close to normal, whereas PA signifies the chronological age corresponding to an average mortality risk within a reference population. Additionally, HD highlights variations in an individual’s physiological condition compared to a healthy standard [10].

Gut flora is an essential component of the human life cycle, playing a key role in the absorption and metabolism of food, the excretion and degradation of toxins, and hormone secretion [11]. Increasing evidence has indicated that the gut microbiota is actively involved in the aging process [12]. The study by Lihao Cheng revealed that by supplementation with the probiotic *Lactobacillus paracasei* PS23, the gut microbiota can be effectively adjusted, oxidative stress and inflammatory responses triggered by aging can be combated, and cognitive function in aged mice can be significantly enhanced [13]. The study by Jingjing Wang showed that gut microbiota plays an important role in slowing down the aging process; for example, *Bacteroides fragilis* in the gut of centenarians can exert an anti-inflammatory effect by upregulating IL-10, which in turn slows the aging process [14].

Diet functions as a critical regulator in maintaining human health [15]. Despite the prevalent focus on the impacts of dietary patterns and components on health, the relationship between diet and gut microbiota remains underexplored. To fill the gap in this field, Susan E. Steck and others identified 14 kinds of food or nutrients related to gut microbiota by reviewing 106 articles. Foods or nutrients that have a positive effect are believed to be able to enhance gut microbial diversity, increase the total content of short-chain fatty acids, or reduce the ratio of Firmicutes to Bacteroidetes, such as fermented dairy products, chickpeas, soybeans, whole grains, fiber, cranberries, avocados, broccoli, coffee, and green tea, and the like fall into this category. Conversely, foods harmful to the health of the gut microbial mainly include red meat, processed meat, refined grains, and a high-fat diet [16]. The team also developed and evaluated a new dietary index for gut microbiota (DI-GM) by using the dietary data of the National Health and Nutrition Examination Survey (NHANES) of the United States. However, there are currently few studies exploring the association between DI-GM and biological age. Since improving health through diet is easier, more cost-effective, and promotes better compliance, it is necessary to explore the association between DI-GM and aging.

Obesity is a global issue that contributes to numerous health conditions, including type 2 diabetes and CVD, posing a significant public health threat [17]. Numerous studies have indicated that maintaining a healthy gut microbiota can aid in weight loss, thereby reducing aging biomarker levels [18,19]. Therefore, this study analyzed data from NHANES 2007–2018 and hypothesizes that the consumption of foods beneficial to the gut microbiota may slow the aging process through weight loss, thus providing novel epidemiological insights into delaying aging.

## 2. Method

### 2.1. Study Design and Participants

This study used data from the NHANES, which covers six cycles from 2007 to 2018. NHANES is a national, dynamic, cross-sectional study employing complex, multistage probability sampling designed to assess the health and nutritional status of the American population [20]. Further details are available on the NHANES official website: https://www.cdc.gov/nchs/nhanes/ (accessed on 30 September 2024).

**Figure 1:**
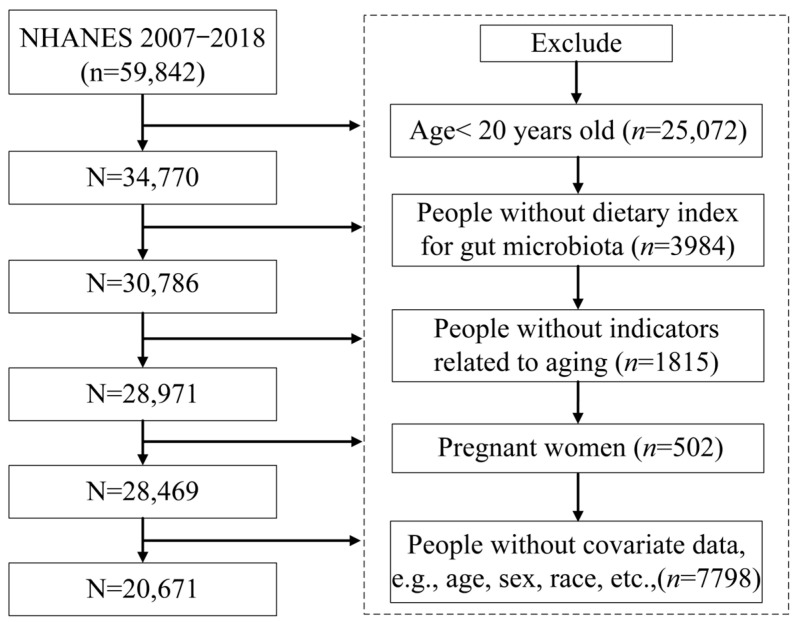
Flowchart of participant selection.

In this study, participants aged 20 years or older were included, while participants under 20 years of age (*n* = 25,072), participants with incomplete data on DI-GM (*n* = 3984), participants without biomarker data related to aging (*n* = 1815), pregnant women (*n* = 502), and participants with missing covariate information, such as demographic information, BMI, HEI, energy intake, smoking status, alcohol status, and disease history (*n* = 7798), were excluded. Finally, 20,671 participants were included in the study. Figure 1 details the inclusion and exclusion criteria.

### 2.2. Outcome Variable

In our study, we employed the ‘BioAge’ R package for computing biological aging, which allowed us to implement various established aging models, including KDM, PA, and HD. The ‘BioAge’ package is a comprehensive tool designed for the calculation of biological age based on quantifiable biomarkers. It integrates multiple algorithms that are widely recognized for their accuracy and reliability in aging research. The website https://github.com/dayoonkwon (accessed on 30 September 2024) provides the relevant algorithms and R code for the ‘BioAge’ package. The blood biochemical indexes were initially trained using data from NHANES III [21]. In this study, 11 blood biomarkers were selected: alkaline phosphatase, total cholesterol, uric acid, albumin, creatinine, glycohemoglobin, white blood cell (WBC) count, lymphocyte percentage, mean cell volume, blood urea nitrogen, and red cell distribution width [22,23,24]. The collection and processing of samples were conducted according to the detailed instructions outlined in the Laboratory/Medical Technologists Procedures Manual of the NHANES. Serum samples were stored at −30 °C under freezing conditions before analysis. The measurement of biomarkers in the serum utilized two types of instruments: Beckman Synchron LX20 and Beckman Coulter UniCel^®^ DxC800 (Beckman Coulter, Brea, CA, USA), in accordance with specific biochemical methods. For example, alkaline phosphatase was measured through enzymatic rate methods, albumin via bichromatic digital endpoint methods, blood urea nitrogen through enzymatic conductivity rate methods, and cholesterol and creatinine utilizing timed-endpoint and Jaffe rate methods, respectively. For uric acid, a timed endpoint method was utilized. The blood cell count was completed using the Beckman Coulter MAXM instrument at the Mobile Examination Centers (MECs). Glycated hemoglobin was measured using high-performance liquid chromatography (HPLC). KDM used regression analysis to measure the specific biomarkers and chronological age of the reference population. PA was developed by analyzing various factors related to mortality risks to estimate death risk [9]. The HD was used to determine the extent to which an individual’s physiological measurements differed from the reference values derived from a young and healthy population [9]. KDM or PA greater than chronological age indicates accelerated aging. HD greater than the median of the population indicates homeostasis disorder [2]. C-reactive protein was excluded from the calculations due to its absence in NHANES 2011–2014. Previous studies have shown a high correlation between aging data computed with and without C-reactive protein [1,22,23]. Moreover, a sensitivity analysis was conducted in our study to explore the association between DI-GM and biological age, including C-reactive protein, which showed no significant changes in the results (see Supplementary Table S3).

### 2.3. Predictive Variables

In NHANES, two dietary recall interviews were conducted. The first evaluation was a 24 h dietary recall interview at the MEC, and the second evaluation was a telephone interview, which recorded the comprehensive and detailed information on participants’ dietary intake in the past 24 h, including all foods and drinks. According to research by Bezawit E. Kase et al., 14 kinds of foods and nutrients were finally included in the DI-GM score, comprising 10 foods that promote gut health and 4 that are detrimental to gut health [16]. For beneficial foods, participants with an intake exceeding the sex-specific median are assigned a value of 1, while those below the median receive a value of 0. In contrast, for unfavorable foods, an intake above the sex-specific median is assigned a value of 0, and a value of 1 is given to those below the median. The DI-GM score is determined by adding the scores from each section, with a range between 0 and 14. A higher DI-GM score indicates a healthier gut microbiota.

### 2.4. Covariance

According to the references, the potential confounding variables that could impact biological aging were taken into account [3,8]. These variables included demographic information such as gender, age, race, educational level (less than high school, high school, and college or above), marital status (never married, married, and widowed/divorced/separated); socioeconomic status assessed via the poverty income ratio (PIR), categorized as low (PIR < 1.0), medium (1.0 ≤ PIR < 3.0), and high (PIR ≥ 3.0); and physical activity was measured in metabolic equivalents (MET) multiplied by minutes [25]. Additionally, we incorporated lifestyle factors such as smoking status and alcohol consumption. Energy intake, blood triglyceride level, body mass index (BMI), and healthy eating index (HEI) were also included. Among them, BMI (kg/m^2^) was divided into three groups based on assessment criteria: <25 (underweight or normal weight), ≥25 to 30 (overweight), and ≥30 (obesity). The HEI is categorized as low (HEI < 50), medium (50 ≤ HEI < 70), and high (HEI ≥ 70) [26]. Furthermore, the analysis considered the history of diseases such as CVD, hypertension, diabetes, and cancer. The diagnostic criteria for diabetes include an HbA1c level greater than 6.5%, fasting blood glucose exceeding 126 mg/dL, medical diagnosis of diabetes, or current insulin use.

### 2.5. Statistical Analysis Methods

Following the guidance provided by the NHANES analysis manual, we analyzed the data of NHANES in six periods from 2007 to 2018 by using the sampling weight and also considering the main sampling units and stratification. In the descriptive analysis of baseline characteristics, continuous variables were expressed as weighted means and standard errors, while categorical variables were denoted by weighted percentages. For continuous variables, we used analysis of variance (ANOVA) to assess the differences in baseline characteristics of participants with different DI-GM scores. The chi-square test was employed to examine the differences among groups for categorical variables.

Logistic regression was used to assess the association between DI-GM and biological aging. The risk of accelerated aging was measured using odds ratio (OR) and a 95% confidence interval (CI). Participants were grouped into Q1 (0–4), Q2 (5), Q3 (6), and Q4 (7–14) based on DI-GM scores. Model 1 did not account for covariates; Model 2 controlled for age, sex, education, race, marital status, PIR, MET, energy intake, and BMI. Model 3 was further adjusted for HEI, blood triglyceride levels, smoking, drinking, diabetes, hypertension, CVD, and cancer, in addition to the adjustments made in Model 2.

Considering the potential nonlinear relationship between DI-GM and biological aging, we conducted an RCS analysis. This method adjusted for all covariates (Model 3). A significance threshold of *p* < 0.05 for the nonlinearity test indicated the existence of a nonlinear relationship. The analysis was performed using the ‘rms’ package in R. Next, we used two segmented logistic regression models based on inflection points to examine the association between DI-GM and biological age.

In this study, the R package “mediation” was used to analyze the mediating effects of BMI in the association between DI-GM and biological age (bootstrap test; 2000 iterations). Additionally, stratified analyses and interaction analyses were conducted to explore the association between DI-GM and biological age in populations with varying characteristics.

Finally, we performed multiple sensitivity analyses. First, we incorporated C-reactive proteins in the calculation of biological age and assessed its association with DI-GM. Second, we excluded individuals over 75 years to prevent the potential confounding effects of excessive aging. Third, individuals with extreme energy intake (<500 kcal/day or >6000 kcal/day) were excluded from the study. Finally, we adjusted the NHANES cycle for the original covariates. All results presented in this study were analyzed using R version 4.3.3. A *p*-value of less than 0.05 was considered indicative of statistical significance.

## 3. Results

### 3.1. Basic Information

This study included a total of 20,671 participants, who were categorized into four groups according to the quartiles of DI-GM. Compared with participants with lower DI-GM scores (Q1), participants with higher DI-GM scores had a lower proportion of aging. In addition, participants with higher DI-GM scores showed higher education levels and a larger proportion of women and non-Hispanic Whites, married individuals, and higher PIR levels. In addition, they had higher HEI scores, lower BMI and triglyceride levels, and a lower prevalence of hypertension and diabetes. Participants’ baseline characteristics are detailed in Table 1.

The Supplement Table S1 results showed that, apart from the total cholesterol, significant differences existed among biological age indicators such as albumin, creatinine, lymphocytes, alkaline phosphatase, WBC count, and so on across different DI-GM levels.

### 3.2. Association of the DI-GM and Biological Age Indicators

**Figure 2:**
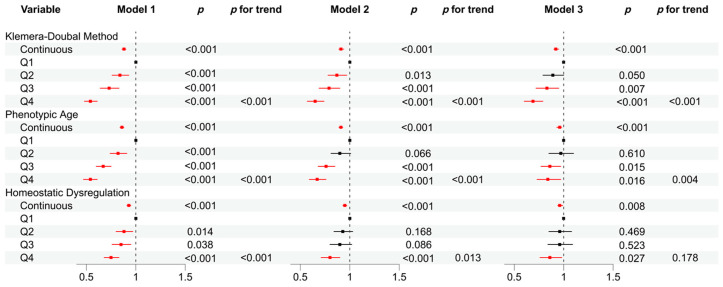
Association between DI-GM and biological age by logistic regression.

Logistic regression results (Figure 2) showed that in Model 1, a higher DI-GM score was negatively associated with an increased risk of KDM, PA, and HD. Compared with Q1, participants in Q2, Q3, and Q4 had a significantly lower risk of higher KDM, PA, and HD. After adjusting for covariates in Model 2, with the increase of DI-GM score, the risk of higher KDM, PA, and HD was also significantly reduced. Compared with Q1, the risk of higher KDM (OR: 0.65, 95% CI: 0.57–0.74), PA (OR: 0.67, 95% CI: 0.59–0.76), and HD (OR: 0.80, 95% CI: 0.71–0.90) in Q4 was significantly reduced by 35%, 33%, and 20%, respectively. In Model 3, as the DI-GM score increased, the risk of higher KDM, PA, and HD remained significantly reduced. Compared with Q1, the risk of higher KDM (OR: 0.69, 95% CI: 0.60–0.79), PA (OR: 0.84, 95% CI: 0.73–0.97), and HD (OR: 0.86, 95% CI: 0.76–0.98) in Q4 decreased by 31%, 16%, and 14%, respectively.

Model 1 did not adjust for covariates;

Model 2 adjusted for age, sex, education, race, marital status, PIR, MET, energy intake, and BMI;

Model 3 was adjusted for age, sex, education, race, PIR, marital status, MET, energy intake, BMI, HEI, blood triglyceride concentration, smoking, drinking, diabetes, hypertension, CVD, and cancer. 

DI-GM, dietary index for gut microbiota. PIR, poverty income ratio. BMI, body mass index. HEI, healthy eating index. MET, metabolic equivalent. CVD, cardiovascular disease.

### 3.3. Non-Linear Trends of the DI-GM and Biological Age Indicators

**Figure 3:**
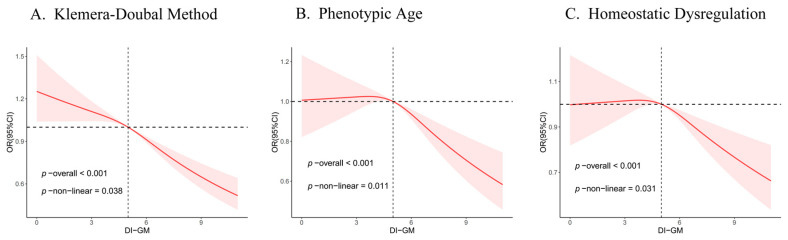
Association between DI-GM and biological age by RCS.

To explore whether there is a nonlinear relationship between DI-GM and biological aging indicators, we employed RCS in our analyses. As depicted in Figure 3, after adjusting for all covariates, with the increase of the DI-GM score, the overall risk of higher KDM, PA, and HD showed a decreasing trend (*p* overall < 0.001). The association between KDM, PA, HD and DI-GM was nonlinear (*p* for non-linear < 0.05). When the DI-GM score was greater than 3.65, the risk of higher PA began to decrease, and when the DI-GM score was greater than 3.76, the risk of higher HD began to decrease. 

Model adjusted for age, sex, education, race, PIR, marital status, MET, energy intake, BMI, HEI, blood triglyceride concentration, smoking, drinking, diabetes, hypertension, CVD, and cancer.

DI-GM, dietary index for gut microbiota. PIR, poverty income ratio. BMI, body mass index. HEI, healthy eating index. MET, metabolic equivalent. CVD, cardiovascular disease. RCS, restricted cubic spline.

We further explored the nonlinear relationships between KDM, PA, and HD with DI-GM, using two segmented logistic regression. Although there was no inflection point for KDM, we chose the point where the OR value was 1 as the inflection point for KDM. The inflection points of KDM, PA, and HD were 5, 3.65, and 3.76, respectively. When DI-GM was ≥ 5, the risk of high KDM decreased by 10% for every additional unit of DI-GM. When DI-GM was ≥ 3.65, the risk of PA decreased by 6%. When DI-GM was ≥ 3.76, the risk of HD decreased by 4% (Supplement Table S2).

### 3.4. Association of the BMI and DI-GM

The results in Supplementary Table S3 showed that in Model 1, higher DI-GM scores were negatively correlated with a higher BMI. Compared with Q1, the β values of DI-GM and BMI in Q4 were −1.79 (−2.17, −1.41) (*p*_for trend_ < 0.05). The trend of Model 2 was similar to that of Model 1. After adjusting for all covariates (Model 3), BMI also showed a significant downward trend with the increase of DI-GM score. Compared with the Q1, the β value of DI-GM and BMI in Q4 was −0.60 (−1.00, −0.20) (*p*_for trend_ = 0.005).

### 3.5. Mediation Analyses

**Figure 4:**
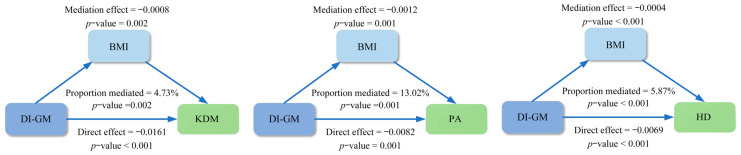
Association of DI-GM and biological age mediated by BMI.

In addition, mediation analyses were performed to assess the mediating mechanisms by which DI-GM affects biological age indicators. The results in Figure 4 showed that BMI had a significant mediating effect on the relationship between DI-GM and biological age indicators. The total effect coefficient of BMI-mediated DI-GM on KDM was −0.0169 (*p* < 0.001), the mediating effect was −0.0008 (*p* = 0.002), and the mediating percentage was 4.73% (*p* = 0.002); the total effect coefficient of BMI-mediated DI-GM on PA was −0.0095 (*p* = 0.001), the mediating effect was −0.0012 (*p* = 0.001), and the mediating percentage was 13.02% (*p* = 0.001); the total effect coefficient of BMI-mediated DI-GM on HD was −0.0073 (*p* < 0.001), the mediating effect was −0.0004 (*p* < 0.001), and the mediating percentage was 5.87% (*p* < 0.001).

Model adjusted for age, sex, education, race, PIR, marital status, MET, energy intake, BMI, HEI, blood triglyceride concentration, smoking, drinking, diabetes, hypertension, CVD, and cancer.

DI-GM, dietary index for gut microbiota. PIR, poverty income ratio. BMI, body mass index. HEI, healthy eating index. MET, metabolic equivalent. CVD, cardiovascular disease. KDM, Klemera–Doubal Method. PA, phenotypic age. HD, homeostasis disorder.

### 3.6. Subgroup Analyses

**Figure 5:**
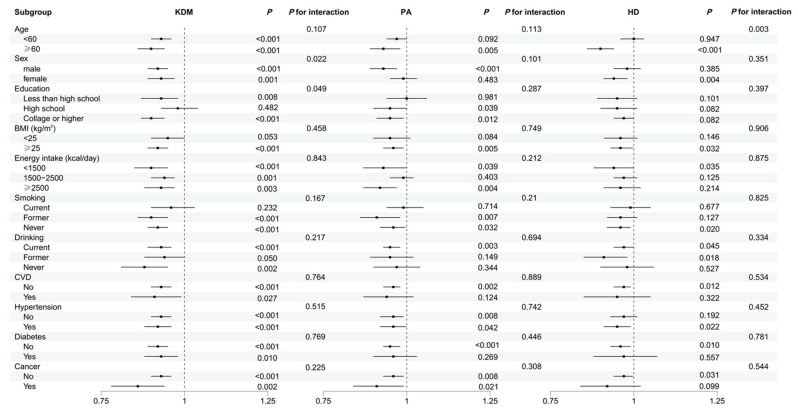
Stratified analysis of the association between DI-GM and biological age.

Participants were stratified according to different characteristics. As shown in Figure 5, there was a significant interaction between DI-GM and sex as well as education level in the association between DI-GM and KDM. Similarly, in the association between DI-GM and HD, there was a significant interaction between DI-GM and age. Moreover, after stratifying the analysis for different subgroups, the association between DI-GM and biological age (KDM, PA, and HD) remained consistent with the main outcomes in patients who were over 60 years old; had a BMI ≥ 25; an energy intake of <1500; never smoked; currently drank alcohol; had no history of CVD, diabetes, or cancer; and in those who had hypertension.

Model adjusted for age, sex, education, race, PIR, marital status, MET, energy intake, BMI, HEI, blood triglyceride concentration, smoking, drinking, diabetes, hypertension, CVD, and cancer.

DI-GM, dietary index for gut microbiota. PIR, poverty income ratio. BMI, body mass index. HEI, healthy eating index. MET, metabolic equivalent. CVD, cardiovascular disease. KDM, Klemera–Doubal Method. PA, phenotypic age. HD, homeostasis disorder.

### 3.7. Sensitivity Analyses

Multiple sensitivity analyses were performed to verify the consistency of the findings. First, C-reactive protein was included in the calculation of biological age indicators. The results showed that in Models 1, 2, and 3, compared with Q1, participants with higher DI-GM scores had a significantly lower risk of higher KDM, PA, and HD, which were consistent with the main study outcomes (see Supplementary Table S4). Furthermore, individuals over 75 years old were excluded to prevent potential confounding effects from excessive aging, and the results remained largely unchanged, showing a negative association between DI-GM and the risk of higher biological age (see Supplementary Table S5). After excluding individuals with extreme energy intake (<500 kcal/day or >6000 kcal/day), the results still showed no significant changes (see Supplementary Table S6). Finally, after adjusting for NHANES cycles, the results were consistent with the primary results (see Supplementary Table S7).

## 4. Discussion

The association between DI-GM and biological age was explored in depth using the NHANES database. We divided the population according to the quartiles of participants’ DI-GM scores. The logistic regression model revealed that the DI-GM score was negatively correlated with higher KDM, PA, and HD. Compared to Q1, the risk of accelerated aging in Q4 was significantly reduced. The RCS analysis further showed the dose–response relationship between DI-GM score and biological age, indicating that the association between DI-GM and biological age was nonlinear. The results of the mediation analysis showed that BMI had a significant mediating effect between DI-GM and biological age. Several sensitivity analyses also confirmed our results.

Biological age is a comprehensive indicator calculated using multiple biomarkers related to the liver, kidney, immune system, and metabolism biomarkers within the human body. It reflects the cumulative effects of multisystemic aging [8]. A new perspective on disease holds that many chronic diseases originate from the gut, and the gut microbiota releases a variety of physiologically active substances into the blood and they enter the circulatory system of the body [27,28]. For example, the liver receives blood from the gut through the portal vein system [29]. Diet can change the gut microbiota of the host in a short time, and then influence overall health [30]. Therefore, consuming more foods that promote gut microbiota is expected to become a new target for maintaining human health. Tarini Shankar Ghosh’s research showed that the Mediterranean diet can further alleviate frailty in the elderly by regulating the composition of specific microbiota in the gut, increasing the content of short-chain/branched fatty acids, reducing the content of secondary bile acids, and reducing the levels of inflammatory markers such as IL-17 and C-reactive protein [31]. Research by Fengjie Huang et al. showed that the brown protein in Pu’er tea can reduce hypercholesterolemia and hyperlipidemia by inhibiting the microorganisms related to the activity of bile salt hydrolase (BSH) in the gut [32]. Sun Yue’s research indicated that probiotic fermented yogurt could increase the content of short-chain fatty acids in the cecum by reshaping the gut microbiota structure. Furthermore, it helped alleviate weight gain, elevated blood lipids, liver lipid metabolism disorders, and fatty degeneration induced by high-fat diets in mice [33]. 

Through a systematic review of 106 articles, Bezawit E. Kase and her team summarized 14 types of foods or nutrients related to gut microbiota and developed the DI-GM. However, the relationship between DI-GM and biological age remains unclear. Our research showed that an increase in the DI-GM score was linked to a significant reduction in the risk of higher KDM, PA, and HD among participants. Similarly, Aimée Parker’s study indicated that in aged mice, fecal transplantation from young mice effectively lowered serum levels of pro-inflammatory cytokines, decelerated inflammation in the central nervous system and retina, and reduced levels of key inflammatory proteins in the retina (such as complement protein C3) by modulating gut microbiota diversity and increasing metabolic products that alleviate aging (acetate and tauro-conjugated bile acids). This effectively reversed signs of aging in the gut, eyes, and brain during the aging process [34]. Research by Junli Ma has shown that transplanting feces from young mice into older mice can enhance the diversity of the microbial communities in the guts of older mice. This improvement in microbial diversity has been linked to better outcomes in liver damage, glucose sensitivity issues, and swelling of the liver and spleen, effectively reversing declines in anti-inflammatory and antioxidant capabilities [35]. These studies, alongside our findings, collectively offer robust support for the significant role of the gut microbiota in mitigating the aging process. 

We discovered that the improvement in DI-GM scores was non-linearly associated with aging. Only when DI-GM scores exceed a specific threshold does the risk of aging begin to decrease. This indicated an important dose–response pattern. This finding emphasizes the importance of making recommendations based on a certain dietary quality and structure, rather than simply increasing or decreasing the intake of specific foods or components.

However, the mechanisms through which DI-GM affects aging require further investigation. Several studies have delved into the factors contributing to aging, which encompass metabolic abnormalities, mitochondrial dysfunction, oxidative stress, and inflammation [2]. A significant finding of our research was the mediating role of BMI in the association between DI-GM and biological age. Numerous studies support the argument that a high BMI accelerates biological aging. For example, a study by Xingqi Cao on the dynamic monitoring of weight from youth to middle age in individuals over 40 years old demonstrated that compared to individuals maintaining normal weight, those whose weight transitioned from overweight to obese, as well as those who remained obese in both periods, exhibit a significantly increased risk of accelerated phenotypic aging [36]. Similarly, research by Shanshan Chen demonstrated that compared to individuals with normal weight, overweight, non-obese to obese, and persistently obese populations exhibit significantly reduced levels of the anti-aging protein biomarker, serum Klotho [37]. Research by Kulapong Jayanama demonstrated that the frailty index in middle-aged and elderly individuals increases significantly with an increase in BMI [38]. The above studies collectively confirmed that being overweight or obese can accelerate the aging process or contribute to a frail condition.

The primary mechanisms that link obesity and aging can be partially explained by changes in metabolic or inflammation [38]. The increase in adipocytes leads to manifestations of a range of metabolic syndromes [38]. Recent research indicated that diet plays a significant role in regulating the body’s metabolism, presenting potential future targets for the treatment of metabolic disorders [39]. A randomized controlled trial conducted by Fathi Y. on premenopausal overweight women demonstrated that blood lipid levels were significantly decreased in the group consuming fermented dairy products compared to the group consuming low-fat milk [40]. Therefore, consuming foods that promote gut health may help reduce lipid metabolism markers by supporting weight loss, ultimately contributing to delayed aging. 

In addition, obesity is a key driver of chronic low-grade inflammation. The adipose tissue in obese individuals can release a series of pro-inflammatory cytokines, including TNF-α and IL-6 [41]. This inflammation is closely related to aging, where long-term chronic inflammation may lead to impaired cell development and a decline in cellular function. Aging cells, in turn, subsequently further aggravate metabolic disorders and inflammation, thus causing degenerative diseases (such as CVD, sarcopenia, cancer, etc.) and accelerating the aging process [42]. Foods beneficial to the gut microbiota play a significant role in modulating the immune system and are involved in the pathophysiological processes of various diseases. Research conducted by Ying-Wei Lan showed that, compared with mice with induced pulmonary fibrosis, the group treated with Kefir yogurt showed a notable downregulation of pro-inflammatory factors such as IL-4, IL-6, and TNF-α [43]. An animal experimental study by Mingmei Li demonstrated that mice in the dietary fiber intervention group showed a significant reduction in body weight and serum pro-inflammatory cytokines compared to mice with liver fibrosis [44]. Consequently, the consumption of foods that promote gut health may mitigate inflammation levels in the body by facilitating weight reduction, thereby decelerating the aging process.

Interaction and stratified analysis play a crucial role in deeply exploring the association between various factors. The findings of this research showed significant differences in the association between DI-GM and HD across different age groups. Specifically, a negative correlation between DI-GM and a higher risk of HD was observed exclusively in individuals over 60 years old. This finding may be due to the decline in intestinal function and immunity in the elderly. Consuming foods that harbor beneficial gut microbiota can help them absorb nutrients more efficiently, enhance immunity, and slow down aging. Nonetheless, for people under 60, their intestinal function and immune system are relatively healthy, hence no significant effect was noticed. 

In addition, DI-GM scores may also be related to educational level, and in populations with higher levels of education, higher DI-GM scores are negatively associated with the risk of aging. This phenomenon might be because people with higher education levels usually also have a higher economic status, allowing them to afford more nutritionally rich food. Furthermore, individuals with higher education are more health-conscious and tend to choose healthier lifestyles [45]. Our research suggested that a higher DI-GM is associated with higher energy intake. This may be because foods that are beneficial for gut health, such as whole grains, legumes, and vegetables, typically have lower energy density, making people feel fuller but requiring more energy intake to meet daily needs. Additionally, a diverse diet structure may lead to an increase in total energy intake. Stratified analysis results showed that in the low energy intake group (<1500 kcal/day), the impact of DI-GM on biological age is consistent with the main results. Inadequate energy intake can lead to a lower metabolic rate, causing the body to start storing energy. In this case, consuming more DI-GM foods can improve nutrient absorption and utilization, enhance immune function, reduce low-grade inflammation, and slow down the aging process [46]. 

The association between DI-GM and aging also differs among populations with different smoking levels; for smokers, even increasing the intake of food beneficial to gut health cannot significantly reduce the risk of aging. This phenomenon may have multiple causes: harmful substances released during smoking, such as nicotine, carbon monoxide, and free radicals, can accelerate oxidative stress, leading to inflammation, DNA damage, and the destruction of key organ functions, which are often irreversible [47]. Additionally, smoking may interfere with the absorption of certain nutrients, such as vitamin C, thus reducing the nutritional value of food [48,49]. Smoking also leads to DNA methylation and telomere shortening, meaning that even increasing the intake of food beneficial to gut health may not repair the damage at the genetic level [50]. In summary, smoking may hinder the effectiveness of a diet promoting gut health in combating aging.

The primary strength of this study lies in its comprehensive analysis of the association between DI-GM and biological age, utilizing a large, nationally representative sample for robust results. Furthermore, the employment of diverse statistical methods has reinforced the stability of the findings. Third, our study concluded that a higher DI-GM score significantly reduces the risk of accelerated aging. This insight may offer potential implications for preventive strategies aimed at delaying aging. 

Nonetheless, the investigation has several limitations. First, data on participants’ food intake were collected through 24 h recall interviews conducted either in a mobile examination center or via telephone interviews, which are susceptible to recall bias. Second, the DI-GM index is an extremely innovative indicator for assessing the quality of diet associated with the gut microbiota, based on a synthesis of intervention studies, and thus possesses a certain level of reliability. However, its calculation does not include foods whose association with the gut microbiome has not been explored. Third, the cross-sectional nature of our study limits its ability to establish temporal relationships or infer causality. While we observed significant associations between DI-GM and biological age indicators, these findings do not imply a directional or causal relationship. The observed associations may be influenced by reverse causation; therefore, this study cannot infer causality. Future research could build upon these findings by utilizing a longitudinal study design and incorporating a broader range of demographics to track changes in biological age over time in response to variations in DI-GM.

## 5. Conclusions

Data from NHANES 2007 to 2018 were utilized to investigate the association between DI-GM scores and biomarkers of biological age such as KDM, PA, and HD. The findings demonstrated that an increase in DI-GM scores significantly reduced the risk of higher KDM, PA, and HD. Furthermore, the association between DI-GM scores and biological age was nonlinear. Notably, BMI partially mediated the association between DI-GM and biological age assessed via KDM, PA, and HD, with mediation proportions of 4.73%, 13.02%, and 5.87%, respectively. However, due to the nature of cross-sectional studies, this study has limitations in inferring causal relationships. Future research should validate these results by using longitudinal research methods to monitor how changes in DI-GM affect the dynamic shifts in biological age over time.

## Figures and Tables

**Table 1:** Basic characteristics of the participants by DI-GM scores.

Variable	Total	Q1 [0, 4]	Q2 [5]	Q3 [6]	Q4 [7, 14]	*p*
Age (years)	48.15 (0.27)	46.89 (0.30)	47.42 (0.36)	48.62 (0.40)	50.70 (0.45)	<0.001
Sex, *n* (%)						<0.001
Female	10,482 (51.09)	3897 (47.46)	2455 (52.57)	1930 (50.45)	2200 (56.38)	
Male	10,189 (48.91)	4227 (52.54)	2324 (47.43)	1851 (49.55)	1787 (43.62)	
Race, *n* (%)						<0.001
Non-Hispanic White	9406 (70.68)	3410 (66.60)	2116 (69.15)	1824 (73.49)	2056 (76.85)	
Non-Hispanic Black	4154 (9.90)	1967 (12.87)	975 (10.15)	660 (8.23)	552 (6.02)	
Mexican	2929 (7.60)	1203 (8.67)	738 (8.54)	539 (7.17)	449 (5.14)	
Others	4182 (11.82)	1544 (11.87)	950 (12.17)	758 (11.11)	930 (11.99)	
Education, *n* (%)						<0.001
Less than high school	4443 (13.66)	2082 (17.19)	1073 (14.43)	730 (11.17)	558 (8.99)	
High school	4720 (22.73)	2130 (27.43)	1133 (23.64)	768 (19.87)	689 (16.24)	
Collage or higher	11,508 (63.60)	3912 (55.38)	2573 (61.93)	2283 (68.96)	2740 (74.77)	
Marital status						<0.001
Married/Partner	12,439 (64.41)	4680 (61.70)	2905 (65.17)	2298 (64.88)	2556 (67.84)	
Never married	3666 (17.35)	1611 (19.35)	834 (17.69)	648 (17.11)	573 (13.76)	
Widowed/Divorced/Separated	4566 (18.24)	1833 (18.95)	1040 (17.14)	835 (18.01)	858 (18.40)	
PIR, *n* (%)						<0.001
Low income	4098 (12.92)	1908 (16.03)	1033 (14.43)	654 (10.87)	503 (7.78)	
Middle income	8672 (35.18)	3692 (39.34)	1953 (34.64)	1551 (33.93)	1476 (29.70)	
High income	7901 (51.90)	2524 (44.63)	1793 (50.93)	1576 (55.20)	2008 (62.52)	
HEI, *n* (%)						<0.001
Low	10,041 (49.05)	5504 (68.67)	2395 (52.94)	1340 (38.12)	802 (20.88)	
Medium	8599 (41.07)	2477 (29.61)	2078 (41.18)	1933 (49.58)	2111 (53.02)	
High	2031 (9.88)	143 (1.73)	306 (5.88)	508 (12.30)	1074 (26.09)	
BMI (kg/m^2^)						<0.001
<25	5679 (28.52)	2068 (25.16)	1260 (27.00)	1056 (29.53)	1295 (35.03)	
25~30	6796 (32.99)	2577 (32.04)	1558 (33.39)	1288 (33.31)	1373 (33.94)	
≥30	8196 (38.49)	3479 (42.80)	1961 (39.61)	1437 (37.16)	1319 (31.03)	
MET	3765.92 (74.52)	4125.44 (111.75)	3812.26 (123.75)	3643.40 (118.59)	3205.58 (105.68)	<0.001
Triglycerides (mg/dL)	152.86 (1.38)	156.66 (1.86)	154.61 (2.70)	152.66 (2.61)	144.58 (2.83)	0.003
Globulin (g/dL)	2.82 (0.01)	2.85 (0.01)	2.83 (0.01)	2.80 (0.01)	2.76 (0.01)	<0.001
Energy intake (kcal/day)	2106.66 (8.78)	2064.32 (11.56)	2095.00 (14.52)	2137.51 (21.77)	2164.40 (14.07)	<0.001
Smoking, *n* (%)						<0.001
Current	4057 (18.50)	1861 (21.65)	970 (19.20)	682 (17.86)	544 (12.88)	
Former	5199 (25.65)	1971 (24.15)	1149 (24.99)	951 (24.82)	1128 (29.74)	
Never	11,415 (55.85)	4292 (54.21)	2660 (55.82)	2148 (57.32)	2315 (57.38)	
Drinking, *n* (%)						0.001
Current	14,640 (77.02)	5678 (75.71)	3338 (75.86)	2690 (77.96)	2934 (79.67)	
Former	3269 (12.78)	1354 (13.33)	762 (13.29)	587 (12.35)	566 (11.70)	
Never	2762 (10.20)	1092 (10.97)	679 (10.85)	504 (9.69)	487 (8.63)	
CVD, *n* (%)						0.210
No	18,369 (91.29)	7133 (90.77)	4257 (91.70)	3385 (91.04)	3594 (91.96)	
Yes	2302 (8.71)	991 (9.23)	522 (8.30)	396 (8.96)	393 (8.04)	
Hypertension, *n* (%)						< 0.001
No	11,719 (61.74)	4496 (59.41)	2695 (61.98)	2196 (63.74)	2332 (63.67)	
Yes	8952 (38.26)	3628 (40.59)	2084 (38.02)	1585 (36.26)	1655 (36.33)	
Diabetes, *n* (%)						<0.001
No	16,726 (85.62)	6404 (83.52)	3894 (85.89)	3109 (87.34)	3319 (87.39)	
Yes	3945 (14.38)	1720 (16.48)	885 (14.11)	672 (12.66)	668 (12.61)	
Cancer, *n* (%)						< 0.001
No	18,595 (89.38)	7387 (90.63)	4318 (89.46)	3381 (88.94)	3509 (87.55)	
Yes	2076 (10.62)	737 (9.37)	461 (10.54)	400 (11.06)	478 (12.45)	
PA, *n* (%)						<0.001
No	13,734 (71.07)	5006 (65.89)	3133 (70.16)	2618 (74.16)	2977 (78.20)	
Yes	6937 (28.93)	3118 (34.11)	1646 (29.84)	1163 (25.84)	1010 (21.80)	
KDM, *n* (%)						<0.001
No	13,342 (65.64)	4869 (60.45)	3044 (64.60)	2522 (67.80)	2907 (73.76)	
Yes	7329 (34.36)	3255 (39.55)	1735 (35.40)	1259 (32.20)	1080 (26.24)	
HD, *n* (%)						<0.001
No	10,336 (56.92)	3822 (53.96)	2373 (57.10)	1948 (57.92)	2193 (60.94)	
Yes	10,335 (43.08)	4302 (46.04)	2406 (42.90)	1833 (42.08)	1794 (39.06)	

DI-GM, dietary index for gut microbiota. PIR, poverty income ratio. BMI, body mass index. HEI, healthy eating index. MET, metabolic equivalent. CVD, cardiovascular disease. KDM, Klemera–Doubal Method. PA, phenotypic age. HD, homeostasis disorder. Continuous variables were expressed as weighted means and standard errors, while categorical variables were expressed as weighted percentages. For continuous variables, the *p*-value was based on the analysis of variance (ANOVA), and for categorical variables, the *p*-value was based on the chi-square test. A higher DI-GM score indicates a healthier gut microbiota.

## Data Availability

The datasets supporting the conclusions of this article are available in the NHANES repository, https://www.cdc.gov/nchs/nhanes/index.htm (accessed on 30 September 2024).

## Supplementary Materials

The following supporting information can be downloaded at: https://www.mdpi.com/article/10.3390/nu16234164/s1, Table S1 The differences in biological age indicators among populations with varying DI-GM scores. Table S2 Threshold effect analysis of DI-GM on biological age. Table S3 Association between DI-GM and BMI. Table S4 Association between DI-GM and biological age (including C-reactive protein to evaluate biological age). Table S5 Association between DI-GM and biological age (excluding participants over 75 years of age). Table S6 Association between DI-GM and biological age (excluding energy intake <500 kcal/day or > 6000 kcal/day). Table S7 Association between DI-GM and biological age (adjusted for NHANES cycle).
