# Supplementary material for: The Mediating Role of Body Mass Index in the Association Between Dietary Index for Gut Microbiota and Biological Age: A Study Based on NHANES 2007–2018
Source: Nutrients. 2024 Nov 30;16(23):4164. doi: 10.3390/nu16234164 (PMC11643866; doi:10.3390/nu16234164)
Supplement: Supplementary file 1 [file nutrients-16-04164-s001.zip › nutrients-3323932-supplementary.pdf]

Table S1. The differences in biological age indicators among populations with varying DI-GM scores.

Variable	Total	Q1 [0,4]	Q2 [5]	Q3 [6]	Q4 [7,14]	*P*
Albumin, g/L	42.72(0.05)	42.53(0.06)	42.69(0.08)	42.86(0.08)	42.96(0.08)	< 0.001
Creatinine, umol/L	78.57(0.29)	80.32(0.56)	77.88(0.45)	78.52(0.58)	76.31(0.43)	< 0.001
Alkaline phosphatase, U/L	67.77(0.28)	69.96(0.38)	67.93(0.53)	66.70(0.47)	64.78(0.46)	< 0.001
Total cholesterol, mg/dL	194.38(0.54)	193.58(0.62)	194.62(1.02)	194.56(0.92)	195.34(1.01)	0.470
Uric acid, mg/dL	5.45(0.02)	5.54(0.03)	5.43(0.03)	5.47(0.03)	5.28(0.03)	< 0.001
Glycohemoglobin, %	5.63(0.01)	5.68(0.01)	5.62(0.02)	5.61(0.02)	5.58(0.02)	< 0.001
WBC count (1000 cells/uL)	7.22(0.03)	7.38(0.04)	7.31(0.06)	7.11(0.05)	6.94(0.05)	< 0.001
Lymphocyte percent, %	30.20(0.11)	29.97(0.16)	30.00(0.15)	30.55(0.19)	30.50(0.18)	0.020
Mean cell volume, fL	89.48(0.10)	89.35(0.12)	89.22(0.13)	89.49(0.14)	89.99(0.14)	< 0.001
Blood urea nitrogen, mg/dL	13.76(0.07)	13.94(0.09)	13.67(0.11)	13.74(0.11)	13.59(0.13)	0.030
Red cell distribution width, %	13.21(0.02)	13.28(0.02)	13.25(0.03)	13.20(0.03)	13.04(0.03)	< 0.001

DI-GM, dietary index for gut microbiota.

Table S2. Threshold effect analysis of DI-GM on biological age.

	Inflection point	OR (95%CI)	*P*
KDM	DI-GM < 5	0.99(0.92,1.06)	0.722
	DI-GM ≥ 5	0.90(0.86,0.95)	<0.001
PA	DI-GM < 3.65	1.06(0.93,1.20)	0.385
	DI-GM ≥ 3.65	0.94(0.90,0.98)	0.003
HD	DI-GM < 3.74	1.11(0.96,1.30)	0.164
	DI-GM ≥ 3.74	0.96(0.92,0.99)	0.017

The model was adjusted for age, sex, education, race, PIR, marital, MET, energy intake, BMI, HEI, blood triglyceride concentration, smoking, drinking, diabetes, hypertension, CVD, and cancer.

DI-GM, dietary index for gut microbiota. PIR, poverty income ratio. BMI, body mass index. HEI, healthy eating index. MET, metabolic equivalent. CVD, cardiovascular disease.

Table S3. Association between DI-GM and BMI.

Variable	Model1		Model2		Model3	
	β(95%CI)	*P* for trend	β(95%CI)	*P* for trend	β(95%CI)	*P* for trend
Continuous	-0.41(-0.49, -0.33)		-0.37(-0.46, -0.29)		-0.14(-0.22, -0.05)	
Q1	Ref		Ref		Ref	
Q2	-0.45(-0.79, -0.11)		-0.39(-0.73, -0.05)		-0.07(-0.39, 0.25)	
Q3	-0.96(-1.32, -0.60)		-0.84(-1.20, -0.49)		-0.17(-0.52, 0.19)	
Q4	-1.79(-2.17, -1.41)	<0.001	-1.64(-2.04, -1.23)	<0.001	-0.60(-1.00, -0.20)	0.005

The model was adjusted for age, sex, education, race, PIR, marital, MET, energy intake, BMI, HEI, blood triglyceride concentration, smoking, drinking, diabetes, hypertension, CVD, and cancer.

DI-GM, dietary index for gut microbiota. PIR, poverty income ratio. BMI, body mass index. HEI, healthy eating index. MET, metabolic equivalent. CVD, cardiovascular disease.

Table S4. Association between DI-GM and biological age (including C-reactive protein to evaluate biological age).

Variable	Model1			Model2			Model3		
	**OR (95%CI)**	***P***	***P* for trend**	**OR (95%CI)**	***P***	***P* for trend**	**OR (95%CI)**	***P***	***P* for trend**
KDM									
Continuous	0.88(0.85,0.91)	<0.001		0.92(0.89,0.95)	<0.001		0.94(0.91,0.97)	<0.001	
Q1	Ref						Ref		
Q2	0.85(0.73,0.99)	0.041		0.91(0.78,1.07)	0.250		0.95(0.80,1.13)	0.583	
Q3	0.72(0.61,0.84)	<0.001		0.80(0.68,0.94)	0.010		0.84(0.71,1.01)	0.058	
Q4	0.57(0.49,0.65)	<0.001	<0.001	0.70(0.60,0.81)	<0.001	<0.001	0.76(0.65,0.89)	<0.001	<0.001
PA									
Continuous	0.85(0.83,0.88)	<0.001		0.90(0.88,0.93)	<0.001		0.95(0.92,0.99)	0.007	
Q1	Ref			Ref			Ref		
Q2	0.80(0.72,0.90)	<0.001		0.89(0.79,1.01)	0.079		0.99(0.85,1.15)	0.890	
Q3	0.68(0.59,0.79)	<0.001		0.77(0.66,0.89)	<0.001		0.87(0.74,1.03)	0.102	
Q4	0.50(0.43,0.58)	<0.001	<0.001	0.63(0.54,0.73)	<0.001	<0.001	0.81(0.67,0.97)	0.021	0.004
HD									
Continuous	0.93(0.90,0.95)	<0.001		0.94(0.91,0.97)	<0.001		0.97(0.93,1.00)	0.056	
Q1	Ref			Ref			Ref		
Q2	0.93(0.82,1.07)	0.302		0.96(0.84,1.10)	0.548		1.03(0.88,1.21)	0.704	
Q3	0.79(0.67,0.92)	0.004		0.80(0.68,0.95)	0.013		0.87(0.73,1.04)	0.121	
Q4	0.72(0.63,0.83)	<0.001	<0.001	0.74(0.63,0.86)	<0.001	<0.001	0.84(0.71,0.99)	0.042	0.017

Model 1 did not adjust for covariates.

Model 2 adjusted for age, sex, education, race, marital status, PIR, MET, energy intake, and BMI.

Model 3 was adjusted for age, sex, education, race, PIR, marital, MET, energy intake, BMI, HEI, blood triglyceride concentration, smoking, drinking, diabetes, hypertension, CVD, and cancer.

DI-GM, dietary index for gut microbiota. PIR, poverty income ratio. BMI, body mass index. HEI, healthy eating index. MET, metabolic equivalent. CVD, cardiovascular disease. KDM, Klemera-Doubal Method. PA, phenotypic age. HD, homeostasis disorder.

Table S5. Association between DI-GM and biological age (excluding participants over 75 years of age).

Variable	KDM			PA			HD		
	**OR (95%CI)**	***P***	***P* for trend**	**OR (95%CI)**	***P***	***P* for trend**	**OR (95%CI)**	***P***	***P* for trend**
Continuous	0.93(0.90,0.96)	<0.001		0.96(0.94,0.99)	0.010		0.97(0.94,1.00)	0.049	
Q1	Ref			Ref			Ref		
Q2	0.89(0.79,1.01)	0.075		0.98(0.86,1.12)	0.777		0.97(0.86,1.09)	0.590	
Q3	0.83(0.72,0.96)	0.014		0.87(0.77,0.99)	0.032		0.97(0.85,1.12)	0.688	
Q4	0.70(0.61,0.81)	<0.001	<0.001	0.88(0.76,1.02)	0.089	0.023	0.89(0.78,1.03)	0.115	0.335

The model was adjusted for age, sex, education, race, PIR, marital, MET, energy intake, BMI, HEI, blood triglyceride concentration, smoking, drinking, diabetes, hypertension, CVD, and cancer.

DI-GM, dietary index for gut microbiota. PIR, poverty income ratio. BMI, body mass index. HEI, healthy eating index. MET, metabolic equivalent. CVD, cardiovascular disease. KDM, Klemera-Doubal Method. PA, phenotypic age. HD, homeostasis disorder.

Table S6. Association between DI-GM and biological age (excluding energy intake <500 kcal/day or > 6000 kcal/day).

Variable	KDM			PA			HD		
	**OR (95%CI)**	***P***	***P* for trend**	**OR (95%CI)**	***P***	***P* for trend**	**OR (95%CI)**	***P***	***P* for trend**
Continuous	0.92(0.90,0.95)	<0.001		0.95(0.93,0.98)	<0.001		0.96(0.94,0.99)	0.009	
Q1	Ref			Ref			Ref		
Q2	0.89(0.79,1.00)	0.047		0.97(0.85,1.10)	0.587		0.95(0.85,1.07)	0.436	
Q3	0.82(0.72,0.94)	0.006		0.86(0.77,0.97)	0.016		0.96(0.84,1.09)	0.478	
Q4	0.69(0.60,0.79)	<0.001	<0.001	0.84(0.73,0.97)	0.016	0.004	0.86(0.76,0.99)	0.031	0.193

The model was adjusted for age, sex, education, race, PIR, marital, MET, energy intake, BMI, HEI, blood triglyceride concentration, smoking, drinking, diabetes, hypertension, CVD, and cancer.

DI-GM, dietary index for gut microbiota. PIR, poverty income ratio. BMI, body mass index. HEI, healthy eating index. MET, metabolic equivalent. CVD, cardiovascular disease. KDM, Klemera-Doubal Method. PA, phenotypic age. HD, homeostasis disorder.

Table S7. Association between DI-GM and biological age (adjusted for NHANES cycle).

Variable	KDM			PA			HD		
	**OR (95%CI)**	***P***	***P* for trend**	**OR (95%CI)**	***P***	***P* for trend**	**OR (95%CI)**	***P***	***P* for trend**
Continuous	0.93(0.90,0.96)	<0.001		0.96(0.94,0.99)	0.011		0.97(0.94,0.99)	0.020	
Q1	Ref			Ref			Ref		
Q2	0.91(0.81,1.02)	0.099		0.99(0.87,1.12)	0.838		0.96(0.86,1.09)	0.549	
Q3	0.84(0.73,0.96)	0.014		0.88(0.78,1.00)	0.049		0.97(0.85,1.10)	0.595	
Q4	0.72(0.63,0.83)	<0.001	<0.001	0.89(0.77,1.04)	0.138	0.037	0.89(0.78,1.01)	0.067	0.272

The model was adjusted for age, sex, education, race, PIR, marital, MET, energy intake, BMI, HEI, blood triglyceride concentration, smoking, drinking, diabetes, hypertension, CVD, and cancer.

DI-GM, dietary index for gut microbiota. PIR, poverty income ratio. BMI, body mass index. HEI, healthy eating index. MET, metabolic equivalent. CVD, cardiovascular disease. KDM, Klemera-Doubal Method. PA, phenotypic age. HD, homeostasis disorder.
